# PSMB4/MHC-I Signaling in the Cerebrospinal Fluid-Contacting Nucleus Mediates Neuroinflammatory Depression in Mice

**DOI:** 10.3390/ijms27114798

**Published:** 2026-05-26

**Authors:** Yi-Jun Zhang, Yu-Wei Ma, Bin Gui, Xin-Ling Wang, Jin Qian, Yu Peng, Li-Cai Zhang

**Affiliations:** 1 Jiangsu Province Key Laboratory of Anesthesiology, Xuzhou Medical University, Xuzhou 221004, China; zhangyijun0709@126.com (Y.-J.Z.);; 2 Jiangsu Province Key Laboratory of Anesthesiology and Brain Science, Xuzhou Medical University, Xuzhou 221004, China

**Keywords:** CSF-contacting nucleus, neuroinflammation, depressive-like behaviors, PSMB4/MHC-I, chemogenetics

## Abstract

Neuroinflammation is increasingly implicated in depression pathogenesis, yet the underlying mechanisms are still unclear. This study explores whether PSMB4/MHC-I signaling in the cerebrospinal fluid (CSF)-contacting nucleus mediates neuroinflammatory depression. A persistent neuroinflammation-associated depression model was established in mice by repeated intracerebroventricular lipopolysaccharide (LPS) administration. Depressive-like behaviors were evaluated using established assays. Neuroinflammatory responses and target protein expression were assessed by immunofluorescence, Western blotting, RT-qPCR, and laser capture microdissection. Neuronal activity was mapped by c-Fos staining and manipulated using chemogenetics, alongside pharmacological and genetic interventions. Repeated LPS administration induced significant depressive-like behaviors and obvious neuroinflammation in the CSF-contacting nucleus. Under these conditions, neuronal activity in this nucleus was selectively enhanced. Crucially, chemogenetic activation of these neurons alleviated depressive phenotypes, whereas their inhibition induced depression. Molecularly, LPS significantly upregulated PSMB4 and MHC-I expression. Pharmacological suppression of upstream neuroinflammation reversed this PSMB4 upregulation, and targeted PSMB4 knockdown reduced MHC-I expression, ultimately ameliorating depressive-like behaviors. These findings identify the CSF-contacting nucleus as a critical node in neuroinflammation-induced depression and reveal a novel PSMB4/MHC-I signaling axis linking central inflammatory responses to behavioral deficits.

## 1. Introduction

Major depressive disorder (MDD) is increasingly linked to neuroimmune dysregulation, particularly in treatment-resistant cases, supporting the concept of inflammation-associated depression [1,2]. Although systemic lipopolysaccharide (LPS) administration induces transient immune-related behaviors in rodents, these effects typically resolve within 24 h and fail to model persistent depression [3]. In contrast, repeated intracerebroventricular (i.c.v.) LPS administration induces sustained central inflammation and long-lasting depressive-like behaviors, providing a more translationally relevant model for mechanistic studies [4].

Previous investigations into neuroinflammation-related depression have primarily focused on classical limbic circuits (e.g., the prefrontal cortex and hippocampus), emphasizing cytokine-mediated neural communication while comparatively neglecting the contribution of broader neuro-humoral regulatory pathways [5,6,7].

The cerebrospinal fluid (CSF)-contacting nucleus is a unique brain structure initially discovered and characterized by our research group, with its presence now confirmed across rats, mice, and macaques [8,9,10]. Its distinct cytoarchitecture features somata located within the brain parenchyma that project widely to multiple functional brain regions [11,12,13,14], while their terminal processes extend directly into the CSF. This structural arrangement enables the nucleus to both sense changes in the CSF microenvironment and release signaling molecules into it, thereby modulating surrounding neural structures bathed in the fluid. Thus, it serves as a critical “bridge” mediating substance transport, signal transmission, and functional regulation at the brain–CSF interface. Previous work from our group has established the vital involvement of the CSF-contacting nucleus in regulating depressive-like behaviors following exposure to psychological stressors, such as restraint stress [15,16]. However, its potential participation in neuroinflammation-associated depression remains unexplored.

PSMB4, a catalytic subunit of the immunoproteasome, has recently emerged from genetic and transcriptomic studies as a candidate risk gene associated with depression [17]. As an essential protein involved in antigenic peptide generation and the regulation of major histocompatibility complex class I (MHC-I) expression, PSMB4 plays a critical role in shaping adaptive immune responses [18]. While PSMB4/MHC-I signaling has been extensively studied in the context of peripheral immunity, viral defense, and neurodegenerative processes, its function within emotion-related neural circuits remains poorly understood. Moreover, its relevance in brain regions with direct immune-sensing capabilities—such as the CSF-contacting nucleus—has never been examined. Given that neuronal MHC-I is increasingly recognized as an important regulator of synaptic plasticity and neuronal excitability [19], its potential involvement in neuroinflammation-induced emotional dysregulation warrants detailed investigation [20].

Based on these considerations, we hypothesized that the CSF-contacting nucleus serves as a critical hub translating neuroinflammatory signals into depressive-like behaviors via the activation of the PSMB4/MHC-I signaling axis. To test this hypothesis, we established a sustained depression model using repeated i.c.v. LPS administration and characterized the ensuing behavioral and neuroinflammatory alterations. We subsequently employed chemogenetic approaches to assess the causal role of CSF-contacting nucleus neuronal activity in these depressive phenotypes. Finally, we examined the expression, regulation, and functional significance of the PSMB4/MHC-I pathway within this nucleus. Ultimately, this study aims to elucidate a previously unrecognized, CSF-contacting nucleus-centered mechanism linking neuroinflammation to emotional disturbances, thereby highlighting the PSMB4/MHC-I pathway at the brain–CSF interface as a potential therapeutic target.

## 2. Results

### 2.1. Repeated Intracerebroventricular LPS Induces Depressive-like Behaviors and Neuroinflammation in the CSF-Contacting Nucleus

**Figure 1:**
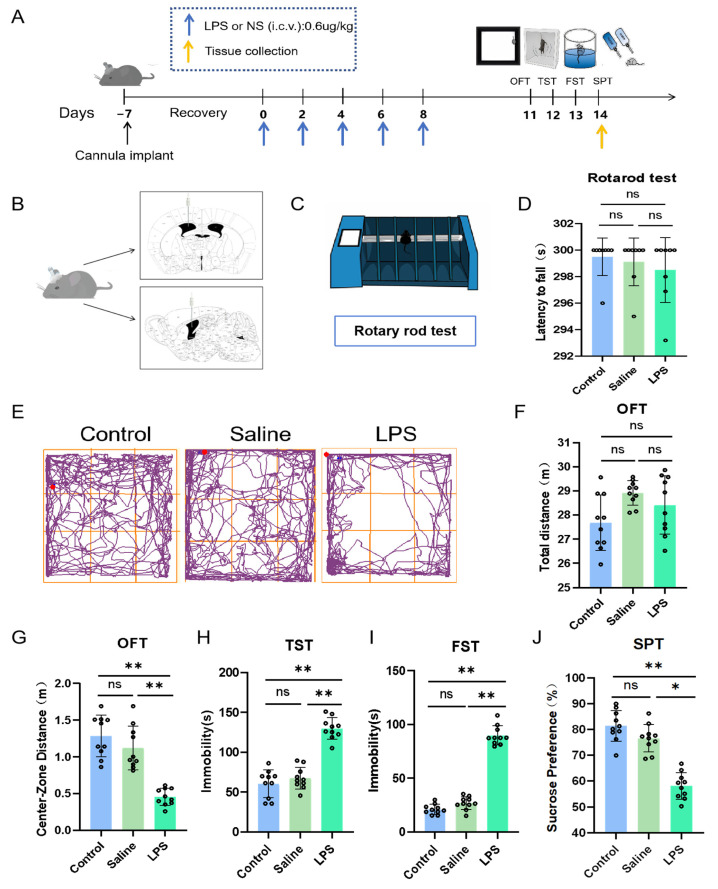
Intracerebroventricular injection of LPS induces depressive-like behaviors in mice. (**A**) Schematic timeline of the experimental protocol. (**B**) Intracranial cannulation site in the mouse brain targeting the lateral ventricle. The coronal brain cross-sectional diagrams are adapted from *The Mouse Brain in Stereotaxic Coordinates* (Paxinos and Franklin, 2nd Edition). (**C**,**D**) Rotarod test (RT) for the assessment of motor coordination and locomotor activity. n = 8 per group. (**E**–**J**) Behavioral assessments of mice in the Control, Saline, and LPS groups. (**E**) Representative movement tracks in the open field test (OFT). (**F**) Total distance and (**G**) center-zone distance in the OFT. (**H**) Immobility time in the tail suspension test (TST). (**I**) Immobility time in the forced swim test (FST). (**J**) Sucrose preference in the sucrose preference test (SPT). In all bar graphs, the blue bars represent the Control group, the light green bars represent the Saline group, and the teal bars represent the LPS group. Data are presented as mean ± SEM (n = 10 per group). * *p* < 0.05, ** *p* < 0.01 (one-way ANOVA followed by Tukey’s test). Abbreviations: CSF, cerebrospinal fluid; RT, rotarod test; OFT, open field test; TST, tail suspension test; FST, forced swim test; SPT, sucrose preference test.

To establish a persistent neuroinflammatory depression model, mice received repeated i.c.v. injections of low-dose LPS (0.6 µg/kg; Figure 1A,B). Behavioral assessment was carried out 72 h after the last injection. Importantly, no significant differences in body weight changes were observed among the groups at this 72 h time point (Figure S1), indicating that the acute sickness-associated systemic effects had largely subsided prior to the behavioral evaluations. Compared with the control group and the saline group, the mice in the LPS treatment group showed a significant decrease in exploration activity in the open field experiment (OFT) (Figure 1E–G), increased motionless time in the suspended tail test (TST) and forced swimming test (FST), and decreased sucrose preference in the sucrose preference test (SPT) (Figure 1H–J), indicating obvious depressive-like behaviors. Statistical analysis revealed no significant variations when comparing the saline and control cohorts. The motor coordination ability evaluated by the rotarod test remained stable between groups (*p* > 0.05), indicating that the behavioral change is not caused by motor function defects (Figure 1C,D).

**Figure 2:**
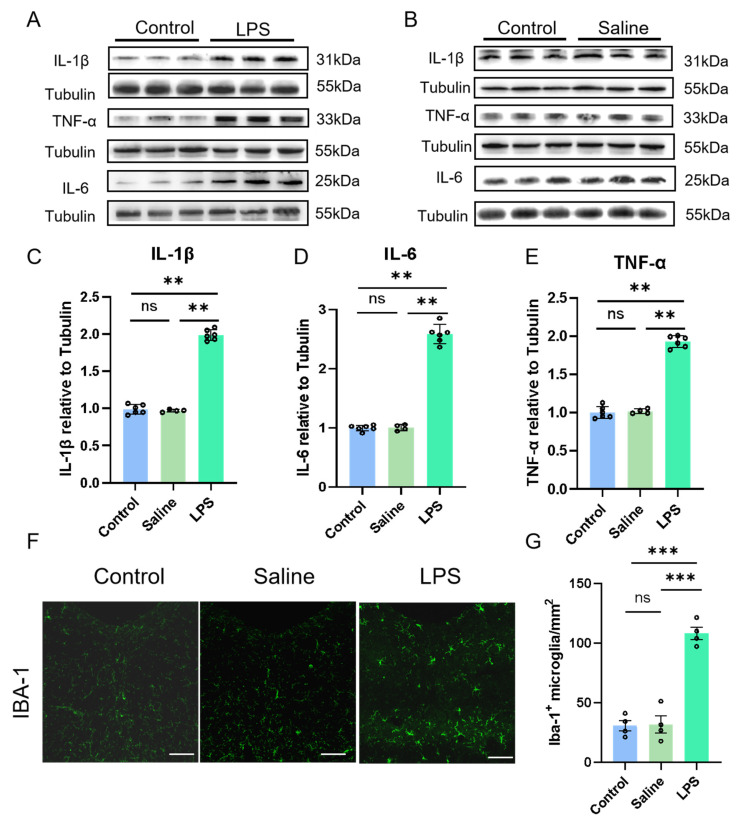
Intracerebroventricular injection of LPS induces neuroinflammation and microglial activation. (**A**,**B**) Representative Western blot images of IL-1β, IL-6, TNF-α, and Tubulin for the Control vs. LPS (**A**) and Control vs. Saline (**B**) groups. (**C**–**E**) Quantitative analysis of relative protein levels of IL-1β (**C**), IL-6 (**D**), and TNF-α (**E**) normalized to Tubulin. (**F**) Representative immunofluorescence images of IBA-1 staining in the CSF-contacting nucleus. (**G**) Quantification of IBA-1^+^ microglia density. In all bar graphs, the blue bars represent the Control group, the light green bars represent the Saline group, and the teal bars represent the LPS group. Data are presented as mean ± SEM (n = 4–6 per group). ** *p* < 0.01, *** *p* < 0.001 (one-way ANOVA followed by Tukey’s test). Abbreviations: CSF, cerebrospinal fluid; IBA-1, ionized calcium-binding adapter molecule 1.

At the molecular and cellular levels, Western blot analysis showed that the expression of TNF-α, IL-1β and IL-6 in the CSF-contacting nucleus of mice treated with LPS was significantly upregulated (Figure 2A–E). Additionally, immunofluorescence staining for IBA-1 demonstrated a substantial increase in microglia density within the same region (Figure 2F,G). These results collectively confirm the successful induction of a robust neuroinflammatory response and microglial activation following chronic LPS challenge.

### 2.2. CSF-Contacting Nucleus Neurons Are Activated Under Neuroinflammation and Modulate Depressive-like Behaviors

**Figure 3:**
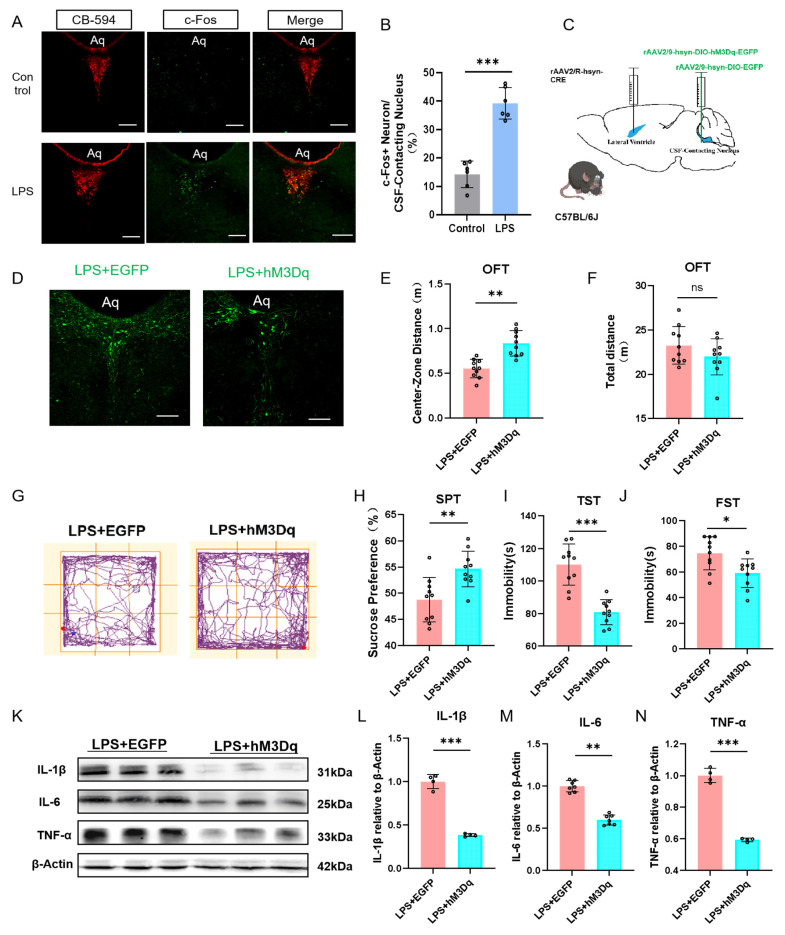
Activation of the CSF-contacting nucleus attenuates LPS-induced depressive-like behaviors and pro-inflammatory cytokine expression. (**A**,**B**) Representative images (**A**) and quantification (**B**) of c-Fos^+^/CB-594^+^ neurons in the Aq. (**C**) Schematic of chemogenetic viral injection. (**D**) Confirmation of EGFP expression. (**E**,**F**) Center-zone distance (**E**) and total distance (**F**) in the OFT. (**G**) Representative movement tracks in the OFT. (**H**–**J**) Sucrose preference in the SPT (**H**), and immobility time in the TST (**I**) and FST (**J**). (**K**) Western blot images of IL-1β, IL-6, TNF-α, and β-Actin. (**L**–**N**) Relative protein levels of IL-1β (**L**), IL-6 (**M**) and TNF-α (**N**). In the immunofluorescence images (**A**,**D**), red indicates CB-594, green indicates c-Fos or EGFP expression, and yellow indicates colocalization. In the bar graph (**B**), the grey bar represents the Control group, and the blue bar represents the LPS group. In the remaining bar graphs, the pink bars represent the LPS+EGFP group, and the cyan bars represent the LPS+hM3Dq group. Data are presented as mean ± SEM. * *p* < 0.05, ** *p* < 0.01, *** *p* < 0.001 (Student’s *t*-test). Abbreviations: CSF, cerebrospinal fluid; Aq, cerebral aqueduct; OFT, open field test; SPT, sucrose preference test; TST, tail suspension test; FST, forced swim test; EGFP, enhanced green fluorescent protein.

To assess neuronal activation, immunofluorescence staining was conducted. LPS treatment increased the expression level of c-Fos in the CSF-contacting nucleus, with levels being significantly higher than that of the control group (Figure 3A,B), indicating selective activation under neuroinflammatory conditions.

In order to evaluate the functional correlation, a dual-virus chemogenetic strategy was adopted. Retrograde AAV-Cre was injected into the lateral ventricles; subsequently, Cre-dependent hM3Dq-EGFP was delivered to the CSF-contacting nucleus (Figure 3C,D). One hour after CNO administration, the activation of these neurons significantly alleviated the depressive-like behaviors in the TST, FST, and SPT (Figure 3E–J), while reducing the LPS-induced TNF-α and IL-1β upregulation (Figure 3K–N).

**Figure 4:**
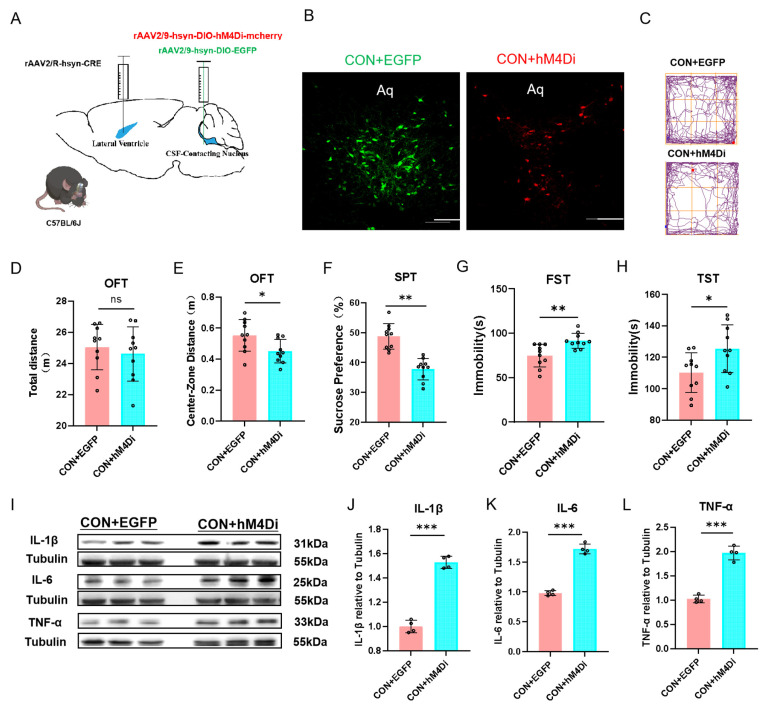
Inhibition of the CSF-contacting nucleus promotes depressive-like behaviors and pro-inflammatory cytokine expression in wild-type mice. (**A**) Schematic of chemogenetic viral injection. (**B**) Confirmation of viral EGFP and hM4Di-mCherry expression in the Aq. (**C**) Representative movement tracks in the OFT. (**D,E**) Total distance and center-zone distance in the OFT. (**F**–**H**) Sucrose preference in the SPT (**F**), and immobility time in the FST (**G**) and TST (**H**). (**I**) Western blot images of IL-1β, IL-6, TNF-α, and Tubulin. (**J**–**L**) Relative protein levels of IL-1β (**J**), IL-6 (**K**), and TNF-α (**L**). In the immunofluorescence images (**B**), green indicates EGFP expression, and red indicates mCherry expression (representing hM4Di). In all bar graphs, the pink bars represent the CON+EGFP group, and the cyan bars represent the CON+hM4Di group. Data are presented as mean ± SEM. * *p* < 0.05, ** *p* < 0.01, *** *p* < 0.001 (Student’s *t*-test). Abbreviations: CSF, cerebrospinal fluid; Aq, cerebral aqueduct; OFT, open field test; SPT, sucrose preference test; FST, forced swim test; TST, tail suspension test; EGFP, enhanced green fluorescent protein.

In order to deeply explore the necessity of this nucleus in emotional regulation, we implemented chemogenetic inhibition (hM4Di) in healthy mice (Figure 4A,B). Silencing of the CSF-contacting nucleus neurons was able to induce depressive-like behaviors (Figure 4C–H) and significantly increase the expression levels of IL-1β, IL-6, and TNF-α (Figure 4I–L).

These findings show that neurons in the CSF-contacting nucleus are activated under neuroinflammatory conditions and play a critical role in modulating depressive-like behaviors.

### 2.3. Repetitive Intracerebroventricular LPS Administration Upregulates PSMB4 and MHC-I Expression in the CSF-Contacting Nucleus

Building on chemogenetic evidence to emphasize the key role of CSF-contacting nucleus neuronal activity in neuroinflammation-induced depressive-like behaviors, we then studied the PSMB4/MHC-I signaling axis. Recent studies have identified that the PSMB4 can be used as a potential therapeutic target for depression [21]. In light of the key role of PSMB4 in antigen processing—facilitating peptide generation and directly regulating MHC-I expression and antigen presentation—we focused on this coordinated signaling pathway. Although previous studies on PSMB4/MHC-I have mainly focused on peripheral immunity and neurodegenerative disorders, its function in depression-relevant brain regions has not been fully explored.

**Figure 5:**
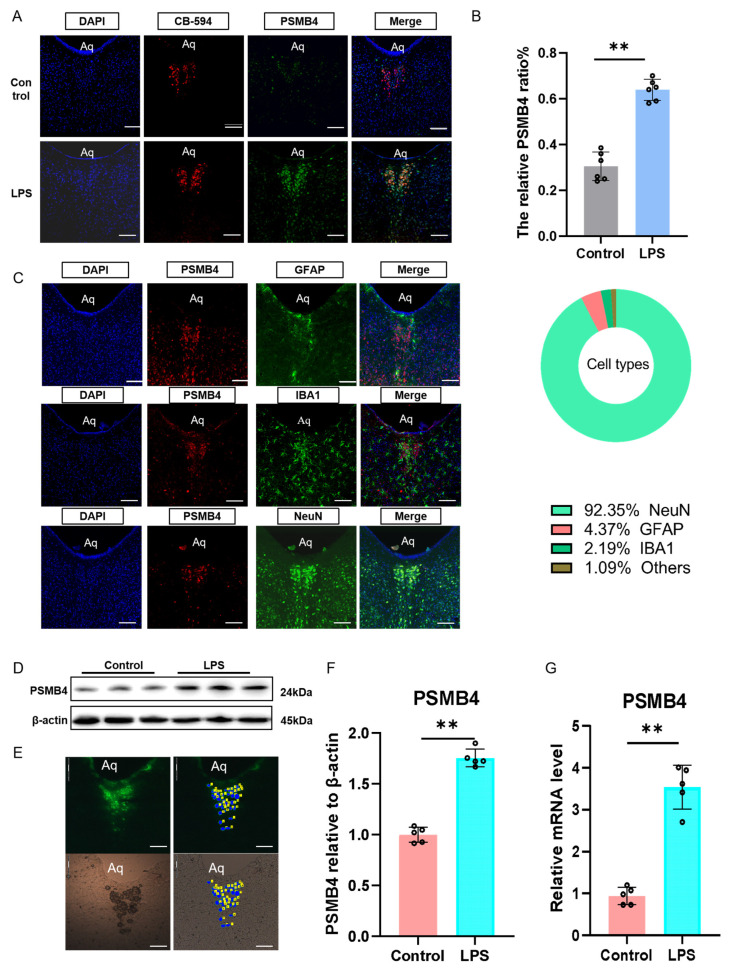
LPS upregulates PSMB4 expression primarily in neurons of the CSF-contacting nucleus. (**A**) Representative immunofluorescence images of PSMB4 and CB-594 in the Aq. (**B**) Quantitative analysis of the relative PSMB4/CB-594 ratio, indicating a significant increase following LPS treatment. (**C**) Representative triple-immunofluorescence staining images of PSMB4 with cell-type-specific markers (NeuN for neurons, GFAP for astrocytes, and IBA1 for microglia). The pie chart illustrates that PSMB4 is predominantly expressed in NeuN-positive cells. (**D**) Representative Western blot bands of PSMB4 and β-actin. (**E**) Representative images of laser capture microdissection (LCM) demonstrating the precise isolation of the CSF-contacting nucleus tissue. (**F**,**G**) Quantitative analysis of the relative protein expression (**F**) and mRNA levels (**G**) of PSMB4 in the isolated tissues. In the immunofluorescence images, DAPI is shown in blue. In panel (**A**), CB-594 is shown in red and PSMB4 is shown in green. In panel (**C**), PSMB4 is shown in red, and the specific cell markers (GFAP, Iba-1, and NeuN) are shown in green. In the bar graphs, the grey bar (**B**) and pink bars (**F**,**G**) represent the Control group, while the light blue bar (**B**) and cyan bars (**F**,**G**) represent the LPS group. Data are presented as mean ± SEM. ** *p* < 0.01 (Student’s *t*-test). Abbreviations: CSF, cerebrospinal fluid; Aq, cerebral aqueduct; IBA1, ionized calcium-binding adapter molecule 1; GFAP, glial fibrillary acidic protein; LCM, laser capture microdissection.

**Figure 6:**
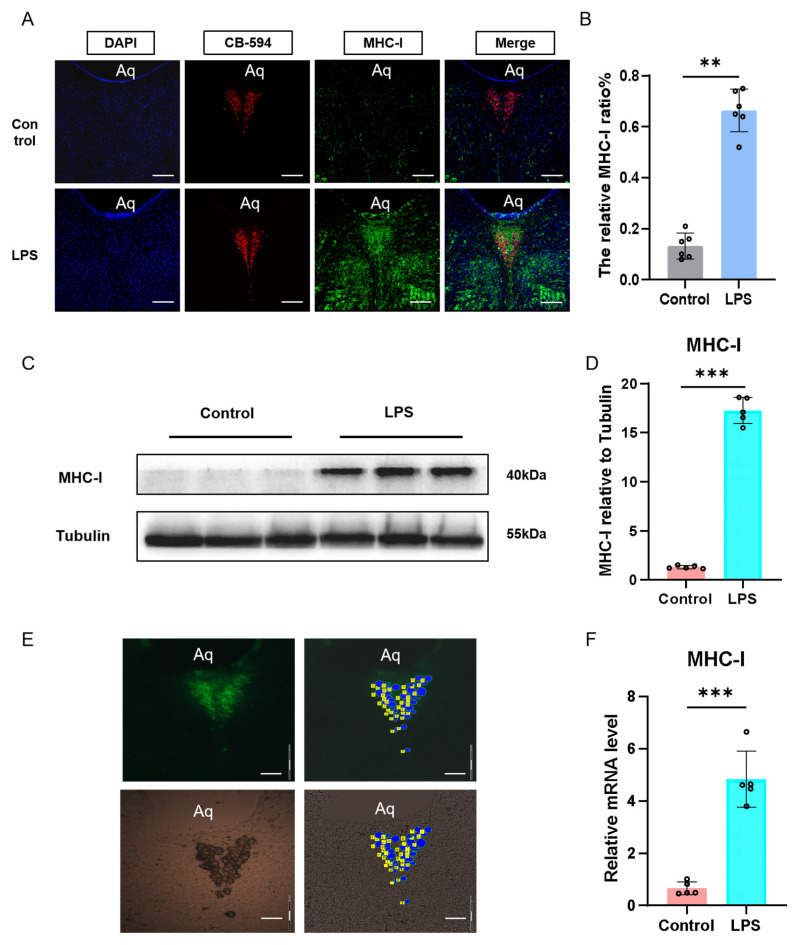
LPS induces the upregulation of MHC-I in the CSF-contacting nucleus. (**A**) Representative immunofluorescence images showing the expression of MHC-I and the CSF-contacting nucleus marker CB-594 in the Aq region. (**B**) Quantitative analysis of the relative MHC-I/CB-594 ratio, indicating a significant increase in MHC-I expression following LPS treatment. (**C**) Representative Western blot bands of MHC-I and the loading control Tubulin. (**D**) Quantitative analysis of the relative protein expression of MHC-I normalized to Tubulin. (**E**) Representative images of laser capture microdissection (LCM) demonstrating the targeted isolation of the CSF-contacting nucleus tissue. (**F**) Quantitative analysis of the relative mRNA levels of MHC-I in the isolated LCM tissues. In the immunofluorescence images (**A**), DAPI is shown in blue, CB-594 is shown in red, and MHC-I is shown in green. In the bar graphs, the grey bar (**B**) and pink bars (**D**,**F**) represent the Control group, while the light blue bar (**B**) and cyan bars (**D**,**F**) represent the LPS group. Data are presented as mean ± SEM. ** *p* < 0.01, *** *p* < 0.001 (Student’s *t*-test). Abbreviations: CSF, cerebrospinal fluid; Aq, cerebral aqueduct; MHC-I, major histocompatibility complex class I; LCM, laser capture microdissection.

We used RT-qPCR and Western blot to evaluate the expression of PSMB4 and MHC-I in the CSF-contacting nucleus under neuroinflammatory conditions. Compared with the control group, the expression of PSMB4 (Figure 5D–G) and MHC-I (Figure 6C–F) of mice in the LPS group increased significantly at the mRNA and protein levels.

In order to determine the cellular distribution of PSMB4, immunofluorescence staining was performed. Analysis showed that PSMB4 was mainly localized in neurons (92.35% of PSMB4^+^ cells co-expressed NeuN), and the expression was extremely low in astrocytes 4.37% GFAP^+^) and microglia (2.19% IBA1^+^) (Figure 5C). Consistent with established anatomical features of the CSF-contacting nucleus, where previous work has shown CB-488-labeled cells to be neuronal [22], we observed robust co-localization of PSMB4 with CB-488^+^ cells under both the control and LPS conditions (Figure 5A). These findings indicate that PSMB4 expression within the CSF-contacting nucleus is predominantly neuronal.

### 2.4. Anti-Inflammatory Minocycline Treatment Reverses LPS-Induced PSMB4 Upregulation

**Figure 7:**
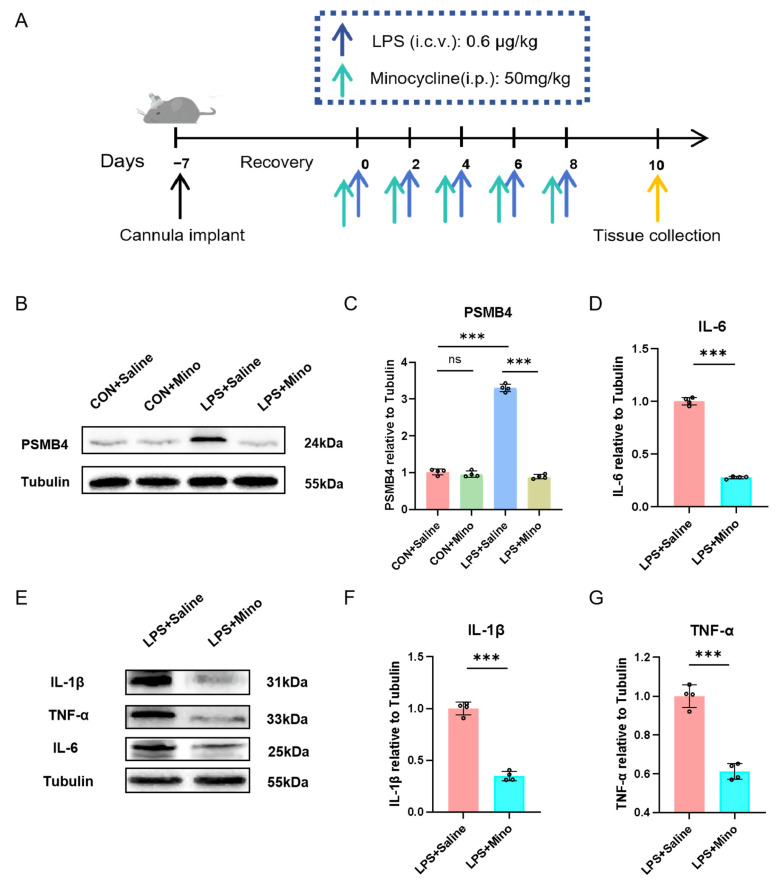
Minocycline attenuates LPS-induced PSMB4 upregulation and neuroinflammation. (**A**) Schematic of the experimental timeline for LPS and Mino administration. (**B**) Western blot images of PSMB4 and Tubulin. (**C**) Relative protein level of PSMB4. (**D**) Relative protein level of IL-6. (**E**) Western blot images of IL-1β, TNF-α, IL-6, and Tubulin. (**F**,**G**) Relative protein levels of IL-1β (**F**) and TNF-α (**G**). In the bar graph (**C**), the pink bar represents the CON+Saline group, the light green bar represents the CON+Mino group, the light blue bar represents the LPS+Saline group, and the olive bar represents the LPS+Mino group. In the remaining bar graphs (**D**,**F**,**G**), the pink bars represent the LPS+Saline group, and the cyan bars represent the LPS+Mino group. Data are presented as mean ± SEM. *** *p* < 0.001 (One-way ANOVA followed by Tukey’s test for C; Student’s *t*-test for **D**,**F**,**G**). Abbreviations: CSF, cerebrospinal fluid; Mino, minocycline.

To investigate whether PSMB4 upregulation is driven by neuroinflammatory signaling, minocycline was administered immediately after each LPS injection according to the experimental timeline (Figure 7A). Western blot analysis further revealed that minocycline [23] significantly suppressed the LPS-induced elevation of IL-1β, IL-6, and TNF-α in the CSF-contacting nucleus, confirming its anti-inflammatory action (Figure 7D–G). Analysis of PSMB4 expression (Figure 7B,C) revealed that LPS significantly increased the PSMB4 protein levels compared to the controls (CON + Saline vs. LPS + Saline); this upregulation was effectively reversed by minocycline treatment (LPS + Saline vs. LPS + Mino). Importantly, the use of minocycline alone did not change the basal PSMB4 expression, as similar profiles were observed in both the CON + Saline and CON + Mino groups.

These results demonstrate that the upregulation of PSMB4 in the CSF-contacting nucleus depends on neuroinflammatory signal transmission and can be reversed by anti-inflammatory intervention, thus establishing PSMB4 as a downstream effector in this inflammatory pathway.

### 2.5. PSMB4 Knockdown in LPS-Treated Mice Reduces MHC-I Expression and Ameliorates Depressive-like Behaviors

**Figure 8:**
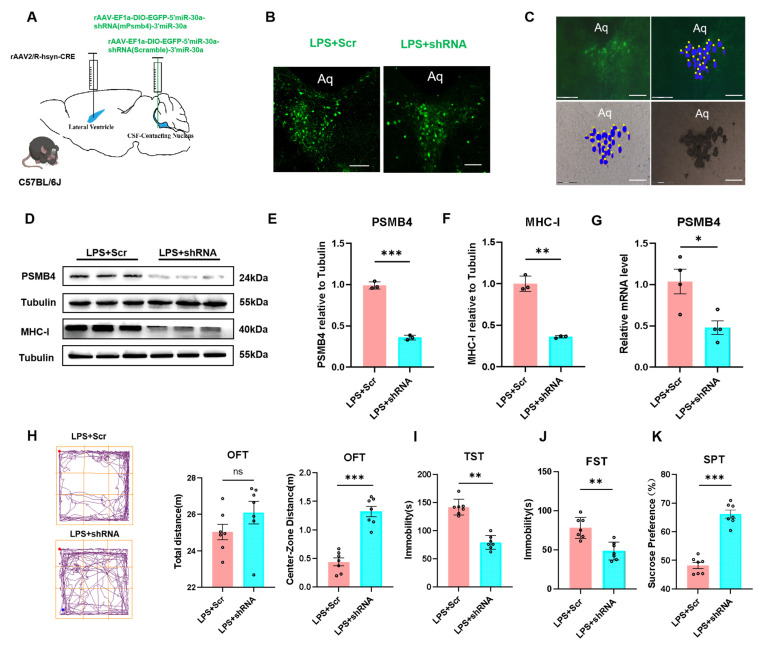
Knockdown of PSMB4 in the CSF-contacting nucleus alleviates LPS-induced depressive-like behaviors and downregulates MHC-I expression. (**A**) Schematic of the viral injection strategy for PSMB4 knockdown (shRNA) in the CSF-contacting nucleus. (**B**) Representative images confirming viral EGFP expression in the Aq. (**C**) Representative images of laser capture microdissection (LCM) targeting the Aq. (**D**) Western blot images of PSMB4, MHC-I, and Tubulin. (**E**,**F**) Relative protein levels of PSMB4 (**E**) and MHC-I (**F**). (**G**) Relative mRNA level of PSMB4. (**H**) Representative movement tracks and quantitative analysis (total distance and center-zone distance) in the OFT. (**I**–**K**) Immobility time in the TST (**I**) and FST (**J**), and sucrose preference in the SPT (**K**). In the immunofluorescence images (**B**,**C**), green indicates EGFP expression. In all bar graphs (**E**–**G**,**H**–**K**), the pink bars represent the LPS+Scr group, and the cyan bars represent the LPS+shRNA group. Data are presented as mean ± SEM. * *p* < 0.05, ** *p* < 0.01, *** *p* < 0.001 (Student’s *t*-test). Abbreviations: CSF, cerebrospinal fluid; Aq, cerebral aqueduct; LCM, laser capture microdissection; MHC-I, major histocompatibility complex class I; OFT, open field test; TST, tail suspension test; FST, forced swim test; SPT, sucrose preference test; EGFP, enhanced green fluorescent protein.

Based on the finding that PSMB4 upregulation is mediated by neuroinflammatory signaling, we further investigated whether PSMB4 knockdown in the CSF-contacting nucleus could alleviate depressive-like behaviors. Using a Cre-dependent viral strategy (Figure 8A), the shRNA-specificity targeting PSMB4 was delivered specifically to the CSF-contacting nucleus. Fluorescence microscopy verified efficient transduction (Figure 8B). Laser capture microdissection realized the accurate collection of transfected neurons (Figure 8C). Both qPCR and Western blot analyses confirmed that PSMB4 was effectively knocked-down at the mRNA (Figure 8G) and protein levels (Figure 8D,E). This knockdown was accompanied by a significant reduction in MHC-I protein expression (Figure 8D,F), confirming that PSMB4 is the upstream regulator of MHC-I in this region. Behavioral assessments revealed that PSMB4 gene knockdown significantly alleviated the depressive behavior in the OFT, TST, FST, and SPT (Figure 8H–K). These research results show that PSMB4 knockdown in the CSF-contacting nucleus suppresses MHC-I expression and alleviates depressive behaviors, indicating that the PSMB4/MHC-I signaling axis is a critical regulator of depression.

## 3. Discussion

In this study, we demonstrated that the CSF-contacting nucleus plays a key regulatory role in depressive-like behaviors induced by neuroinflammation, and we identified the PSMB4/MHC-I signaling axis as a critical inflammatory reactive mediator within this nucleus. We employed intracerebroventricular (i.c.v.) injection of lipopolysaccharide (LPS) to establish a central inflammation model. Compared to psychological stress models like chronic social defeat stress (CSDS), LPS—a potent inducer of neuroimmune cascades—effectively isolates complex neuroendocrine interferences, allowing us to focus precisely on direct “central immune-neuron” interactions. Importantly, our behavioral testing was performed after the acute sickness phase had subsided. Our results demonstrate that mice exhibited persistent depressive-like phenotypes even after acute inflammation subsided, accompanied by significant upregulation of pro-inflammatory cytokines within the CSF-contacting nucleus. Unlike peripheral immune challenges, which typically trigger transient, sickness behavior-dominated hypoactivity that resolves upon cytokine clearance [24,25], the enduring depressive-like phenotype observed here suggests that primary central inflammation exerts a more prolonged pathological remodeling effect on emotional centers. This temporal and phenotypic discrepancy stems fundamentally from the differing mechanisms of immune–brain signaling between central and peripheral challenges. By bypassing the physical restriction of the blood–brain barrier (BBB) and circumventing the rapid endotoxin tolerance typically developed by peripheral immune cells [26], i.c.v administration directly exposes the central microenvironment to immune provocation, initiating a self-amplifying central cascade that operates independently of the periphery. Consistent with these mechanisms, existing literature utilizing repeated central inflammatory paradigms has also reported relatively prolonged neuroinflammatory and depressive-like behavioral alterations compared with classical systemic LPS models. This approach bypasses the blood–brain barrier and has been established in the previous literature to induce chronic microglial hyperactivation and depressive-like deficits that can persist for several weeks, or even months, following the final injection [27].

Existing research on the pathogenesis of neuroinflammation-induced depression has predominantly focused on classical emotional circuits, such as the prefrontal cortex, hippocampus, and amygdala, where pro-inflammatory cytokines disrupt synaptic plasticity and perturb monoamine metabolism [28,29,30]. However, the relationship between the CSF-contacting nucleus and depression has remained unexplored. Distinct from the relatively closed microenvironments of deep parenchymal nuclei, the CSF-contacting nucleus exhibits marked anatomical specialization: its neuronal somata reside within the brain parenchyma, while their terminal processes extend directly into the CSF. This unique topography endows the CSF-contacting nucleus with specialized chemosensory capabilities, positioning it as an active sentinel at the brain–CSF interface [31]. This structural configuration allows the nucleus to continuously monitor the biochemical milieu of the ventricular system. Consequently, when the lateral ventricle is exposed to an immune challenge, these protruding terminals are highly accessible to the locally enriched inflammatory mediators (e.g., IL-1β, TNF-α, and IL-6), making the nucleus exquisitely responsive to neuroimmune fluctuations. It is worth noting that a recent study further confirmed that activation of the CSF-contacting nucleus can exert anti-inflammatory effects in systemic inflammation induced by sepsis, which supports its functional role in modulating neuro-immune crosstalk [32]. Chemogenetic (DREADDs) manipulations further validated the pathophysiological function of the CSF-contacting nucleus. Specific activation of these neurons significantly ameliorated depressive-like phenotypes under neuroinflammatory conditions. Conversely, targeted inhibition of its basal activity in healthy mice was sufficient to induce typical depressive-like behaviors and upregulate pro-inflammatory cytokines. These bidirectional interventions demonstrate that the CSF-contacting nucleus actively translates aberrant neuroimmune signaling into macroscopic affective behavioral outputs, establishing it as a functionally important neuroimmune interface between cerebral homeostasis and complex emotional regulation. Nevertheless, translating localized inflammatory responses at the CSF–brain interface into global behavioral states likely involves complex neural network dynamics. To fully delineate this process, our future research will focus on integrating comprehensive spatial-temporal activity mapping (e.g., time-course c-Fos profiling), and precise circuit-level analyses will be essential. These investigations will clarify how inflammatory signals propagate from the CSF-contacting nucleus to interconnected limbic networks as neuroinflammation progresses.

Our data further highlight a critical role of the PSMB4/MHC-I signaling axis within the CSF-contacting nucleus on neuroinflammation-associated depressive-like behaviors. PSMB4, a catalytic core subunit of the immunoproteasome, has been primarily studied in peripheral immune cells, where it participates in antigen processing and regulates MHC-I expression [33,34]. Recent evidence shows that PSMB4 may be a potential therapeutic target for depression [35,36]. However, its regulation and functional relevance within defined neuronal populations are still rarely studied. In this model, we observed that neuroinflammatory stimulation was associated with a coordinated upregulation of PSMB4 and downstream MHC-I in neurons of the CSF-contacting nucleus.

These research results show that neurons under inflammatory stress may not only act as passive targets for immune signals, but may also actively participate in components of adaptive immune mechanisms to reshape the local immune microenvironment. This view is supported by more and more evidence that neurons can express immune-related molecules, including MHC-I, and participate in immune–neural crosstalk under both physiological and pathological conditions [37,38]. Supporting this view, the expression of PSMB4 within the CSF-contacting nucleus was predominantly neuronal, with approximately 90% co-localization with NeuN and minimal expression in astrocytes or microglia. This neuronal specificity is consistent with previous anatomical characterizations of CB-488-labeled cells in the CSF-contacting nucleus, which have been proven to represent a distinct neuronal population directly interfacing with the cerebrospinal fluid [39]. These findings jointly support the role of the neuronal-centered PSMB4/MHC-I signaling axis in neuroinflammatory-related emotional disorders.

Crucially, our targeted knockdown experiments provide direct causal evidence for this neuron-centric mechanism. Knockdown of PSMB4 using shRNA not only suppressed downstream MHC-I upregulation but also substantially alleviated LPS-induced depressive-like behaviors, confirming that the PSMB4/MHC-I axis drives the pathogenesis. Having established the pathogenic role of neuronal PSMB4, our pharmacological data further elucidate its upstream triggers. Furthermore, systemic minocycline intervention reversed the inflammation-associated upregulation of PSMB4, providing pharmacological evidence that upstream inflammatory signaling drives PSMB4 expression. This aligns with minocycline’s established anti-inflammatory and antidepressant effects in preclinical models [40]. Together, these findings deepen our understanding of the neuroimmune mechanisms underlying depression and indicate that targeting immune-related signaling in specific neural circuits is a promising therapeutic strategy.

While the present study demonstrates that suppression of the PSMB4/MHC-I signaling axis alleviates depressive-like behaviors, the precise neuronal mechanisms downstream of MHC-I remain to be fully elucidated. Emerging evidence suggests that neuronal MHC-I is not merely an immune-related molecule, but also an important regulator of synaptic function and neuronal excitability. Previous studies have shown that elevated neuronal MHC-I can impair NMDA receptor-dependent synaptic plasticity, alter glutamatergic transmission, and influence ion channel dynamics, ultimately affecting neuronal firing properties and circuit activity [41,42]. Furthermore, MHC-I signaling may facilitate neuroimmune interactions through the engagement of immune-associated receptors such as PirB, further amplifying inflammatory neuronal dysfunction [43,44]. In the context of the CSF-contacting nucleus, neuroinflammation-induced MHC-I upregulation may therefore disrupt the intrinsic excitability and synaptic signaling of these neurons, thereby contributing to maladaptive emotional regulation. In this framework, chemogenetic activation of CSF-contacting nucleus neurons may partially compensate for inflammation-associated functional disturbances by enhancing neuronal activity under neuroinflammatory conditions. Neuronal activation was also accompanied by reduced pro-inflammatory cytokine expression, suggesting a potential association between neuronal activity and altered inflammatory responses within the CSF-contacting nucleus. Future studies combining electrophysiological recordings and synaptic functional analyses will be required to directly determine how the PSMB4/MHC-I axis modulates the activity of CSF-contacting nucleus neurons. Additionally, as this study exclusively utilized male mice, future validation in dual-sex cohorts is necessary to address potential sex differences [45].

Beyond these mechanistic explorations, the clinical implications of identifying the neuronal PSMB4/MHC-I axis are particularly noteworthy. Currently, a substantial subset of patients with major depressive disorder who exhibit elevated peripheral and central inflammatory markers remain refractory to conventional monoaminergic antidepressants (e.g., SSRIs). This clinical resistance highlights the inadequacy of solely targeting neurotransmitter reuptake when the underlying pathology is neuroimmune-driven. By delineating the immunoproteasome as a critical intracellular gear that translates neuroimmune disturbances into behavioral deficits, our study pinpoints a more fundamental therapeutic target. Modulating PSMB4 could potentially halt pathological glia–neuron crosstalk at its source within specific emotional hubs, offering a precision-medicine approach for the inflammatory subtype of depression.

In summary, this study establishes the CSF-contacting nucleus and its neuron-specific PSMB4/MHC-I axis as a critical pathogenic link between central neuroinflammation and depressive-like behaviors. These findings enrich our understanding of depression neuroimmunology and suggest that developing CSF-delivered immunoproteasome inhibitors could bypass blood–brain barrier constraints, providing a novel translational strategy for treatment-resistant mood disorders.

## 4. Materials and Methods

### 4.1. Animals

Experiments utilized male C57BL/6 mice (8–10 weeks old, SPF grade). The animals were raised in an environment of 23–25 °C, maintaining a cycle of 12 h of light and 12 h of darkness, and free intake of standard feed and drinking water. All procedures were approved by the Animal Ethics Committee of Xuzhou Medical University (202403T024).

### 4.2. Inclusion/Exclusion Criteria and Humane Endpoints

The animal exclusion criteria, established a priori, were:

1. Unsuccessful i.c.v. cannula placement (post-mortem ink test verification);
2. Severe post-operative complications (>20% weight loss, signs of distress/infection);
3. Meeting humane endpoint criteria before study completion.

### 4.3. Establishment of the Neuroinflammatory Depression Model

Mice were randomly allocated to treatment groups using block randomization to ensure equal group sizes over time. Pentobarbital sodium (1.0%, 0.1 g/kg, i.p.) was used to anesthetize mice, then fix it in the stereo positioning frame. A 27-gauge guide cannula (RWD) was implanted into the lateral ventricle (coordinates: AP −0.6 mm, ML ±1.4 mm, DV −2.10 mm) and fixed with dental bone cement. Following a 7-day recovery period, drug infusions were administered using a microinfusion pump (RWD). Lipopolysaccharide (LPS; Sigma-Aldrich, St. Louis, MO, USA) was dissolved in sterile saline and administered via intracerebroventricular injection at a dose of 0.6 µg/kg [46,47]. Briefly, an internal cannula was inserted, and 1 µL of the LPS solution or sterile saline (for control animals) was infused into the lateral ventricle at a constant rate of 1 µL/min. The cannula was left in place for 10 min after infusion to allow for adequate diffusion. Injection procedure was performed at 9:00 a.m. on alternate days for a total of five administrations (Days 0, 2, 4, 6, and 8). Behavioral testing commenced on Day 11, following the completion of the inflammatory challenge.

### 4.4. Assessment of Depressive-like Behaviors

#### 4.4.1. Open Field Test

Mice were introduced into a square chamber (50 × 50 × 40 cm) for free exploration. A video tracking system recorded locomotor activity, including total distance traveled, number of central zone entries, and center-zone exploration time. Between trials, the chamber was wiped down with 75% ethanol to eliminate odor cues [48].

#### 4.4.2. Forced Swimming Test

Mice were placed in a transparent tank (50 cm high, 20 cm in diameter) filled with water maintained at 23–25 °C. Following an initial 2-min habituation period, the time spent immobile was recorded for the subsequent 4 min [49]. Both the water and the tank were replaced and cleaned between trials to minimize olfactory cues.

#### 4.4.3. Tail Suspension Test

Mice were suspended 30 cm above the surface by adhesive tape affixed to their tails. Behavioral activity was recorded for 6 min, and immobility time—defined as the absence of limb or body movement—was measured during the last 4 min. The apparatus was cleaned with 75% ethanol between trials [50].

#### 4.4.4. Sucrose Preference Test

Mice were first habituated for 24 h to two bottles containing either 1% sucrose or plain water, with bottle positions switched after 12 h. Following 24 h of deprivation, they were given access to both bottles again for a 24-h test period [51]. Sucrose preference, expressed as a percentage, was derived from the formula:

Sucrose preference = (sucrose intake/total fluid intake) × 100%.(1)

#### 4.4.5. Rotarod Test

To rule out potential motor deficits, balance and coordination were measured using an accelerating rotarod (4–40 rpm over 300 s) [52]. The latency to fall was recorded across three trials per mouse. A maximal score was assigned to mice that remained on the rod for the full 300 s. The apparatus was cleaned between trials [53].

### 4.5. CSF-Contacting Nucleus Labeling

Under pentobarbital sodium anesthesia (1.0%, 0.1 g/kg, i.p.) with corneal protection, stereo-positioning injection of mice was conducted by connecting the glass micro-strap of the micro-injection pump (RWD Life Science; St. Louis, MO, USA). The pipette remained in situ for 10 min after injection prior to withdrawal. Stereotaxic coordinates (Paxinos and Franklin atlas) were as follows: lateral ventricle (LV): AP −0.60 mm, ML ±1.40 mm, DV −2.10 mm; CSF-contacting nucleus: AP −5.02 mm, ML 0.00 mm, DV −3.25 mm.

For anatomical tracing, 200 nL of CB-Alexa Fluor 594 was injected into the LV (placement verified by CSF aspiration) [54]. Mice were perfused 5 days later for immunofluorescence visualization of CB-488-labeled CSF-contacting nucleus neurons.

### 4.6. Targeted Knockdown of PSMB4 in the CSF-Contacting Nucleus

For PSMB4 knockdown, a dual-virus strategy was used:

- Silencing group: 200 nL of rAAV-EF1α-DIO-EGFP-5′miR-30a-shRNA(mPSMB4)-3′miR-30a(AAV9, 5.07 × 10^12^ vg/mL, Braincase, Shenzhen, China) was injected into the CSF-contacting nucleus using a microinjector.
- Control group: 200 nL of rAAV-EF1α-DIO-EGFP-5′miR-30a-shRNA(Scramble)-3′miR-30a(AAV9, 5.20 × 10^12^ vg/mL, Braincase, Shenzhen, China) was injected into the CSF-contacting nucleus using a microinjector.

For all groups, 600 nL of rAAV-hSyn-SV40 NLS-Cre (AAV/retro, 1.01 × 10^13^ vg/mL, Braincase, Shenzhen, China) was injected into the LV using a microinjector.

### 4.7. Chemogenetic Strategy for Regulating Activity of the CSF-Contacting Nucleus

The targeting specificity relies on the physical barrier of the lateral ventricle (LV) wall. Because the CSF-contacting nucleus uniquely extends its terminals directly into the CSF, it selectively takes up the virus, whereas off-target transduction in other non-CSF-contacting brain regions is physically prevented.

- Activation group: 200 nL of rAAV2/9-hSyn-DIO-hM3Dq-EGFP (Braincase, Shenzhen, China) was injected into CSF-contacting nucleus using a microinjector.
- Inhibition group: 200 nL of rAAV2/9-hSyn-DIO-hM4Di-mCherry (Braincase, Shenzhen, China) was injected into the CSF-contacting nucleus using a microinjector.
- Control group: 200 nL of rAAV2/9-hSyn-DIO-EGFP (Braincase, Shenzhen, China) was injected into the CSF-contacting nucleus using a microinjector.

For all groups, 600 nL of rAAV-hSyn-SV40 NLS-CRE (AAV/retro, Braincase, Shenzhen, China) was injected into the LV using a microinjector. CNO (GC10822; Montclair, CA, GlpBio, USA) was first dissolved in DMSO to prepare a stock solution at a concentration of 3.33 mg/mL. This stock was subsequently diluted with PBS to obtain a working solution of 0.333 mg/mL (final DMSO concentration: 5%). Mice were administered CNO via intraperitoneal (i.p.) injection at a dose of 3.33 mg/kg (injection volume: 10 mL/kg) 1.5 h before behavioral testing on Day 14 post-injection.

### 4.8. Drugs and Reagents

Drugs and reagents used in this study included CB-Alexa Fluor 594 (Invitrogen, Thermo Fisher Scientific, Waltham, MA, USA), lipopolysaccharide (LPS, Escherichia coli O55:B5; Sigma-Aldrich, St. Louis, MO, USA), minocycline (HY-17412; MCE Shanghai, China), and clozapine-N-oxide (GC10822; Montclair, CA, GlpBio, USA). LPS was dissolved in sterile PBS (0.1 µg/µL) and administered intracerebroventricularly (150 nL per mouse). CNO was dissolved in DMSO (0.2 µg/µL) and injected intraperitoneally (0.2 mL). Minocycline was dissolved in PBS (10 mg/mL) and injected into the abdominal cavity at a dose of 10 mL/kg. All reagents were freshly prepared on the day of use.

### 4.9. Brain Protein Extraction and Western Blot Analysis

To extract total protein, samples from the CSF-contacting nucleus were lysed in RIPA buffer (Beyotime, Shanghai, China) supplemented with protease inhibitors. The tissue homogenates were then centrifuged at 12,000 rpm (4 °C) for 15 min to collect the supernatant. Following quantification via a BCA protein assay, equivalent quantities of protein lysates were resolved by 10% SDS-PAGE. The separated proteins were subsequently electrotransferred to PVDF membranes, which were then blocked with 5% non-fat milk. Overnight incubation at 4 °C was performed with specific primary antibodies against: IL-1β (1:1000, HA601036; HUABIO, Woburn, MA, USA), IL-6 (1:1000, EM1701-45; HUABIO), TNF-α (1:1000, 17590-1-AP; Proteintech, Rosemont, IL, USA), PSMB4 (1:1000, A05105-1; BOSTER, Pleasanton, CA, USA), HLA-ABC (1:1000, HA723415; HUABIO), β-Tubulin (1:10,000, 80713-1-RR; Proteintech), and Actin (1:10,000, 66009-1-Ig; Proteintech). Following washes, the membranes were incubated with HRP-conjugated goat anti-rabbit or anti-mouse secondary antibodies (1:5000, A0208, A0216; Beyotime) at room temperature for 1 h. Finally, the protein bands were visualized using an enhanced chemiluminescence (ECL) detection kit (P0018S, Beyotime). Signals were detected using an Alliance Q9 Advanced imaging system and quantified with ImageJ (Version 1.52a; National Institutes of Health, Bethesda, MD, USA). All Western blot samples represent individual biological replicates, with each sample extracted from a single animal.

### 4.10. Immunofluorescence Staining

Following transcardial perfusion with 4% PFA, brains were post-fixed and cryoprotected in 30% sucrose. Coronal sections (30 µm) of the CSF-contacting nucleus were cut on a cryostat. Tissue slices were permeabilized using 0.1% Triton X-100 and subsequently blocked with 10% donkey serum to prevent non-specific binding. Following this preparation, the sections were subjected to overnight incubation at 4 °C with specific primary antibodies targeting rabbit anti-PSMB4 (1:1000, A05105-1; BOSTER), rabbit anti-HLA-ABC (1:1000, HA723415; HUABIO), rabbit anti-c-Fos antibody (1:400, 2250S; Cell Signaling Technology), mouse anti-NeuN (1:50, HA601111; HUABIO), mouse anti-GFAP (1:500, EM140707; HUABIO), or mouse anti-IBA-1 (1:1000, OB-MMS039; Oasis Biofarm, Hangzhou, China). Corresponding Alexa Fluor 594-conjugated donkey anti-rabbit (1:500, A21207; Abcam, Cambridge, UK) and Alexa Fluor 405-labeled donkey anti-mouse (1:600, ab175658; Abcam, Cambridge, UK) secondary antibodies were applied for 3 h at room temperature. Images were acquired using an Olympus FV1000 confocal microscope (Olympus Corporation, Tokyo, Japan).

Microglial activation was quantified by counting IBA-1-positive cells in the CSF-contacting nucleus across 3–5 sections per animal under identical imaging settings. Cell density was expressed as cells/mm^2^. All analyses were performed blinded using ImageJ.

### 4.11. Tissue Processing and Laser Capture Microdissection

Mice were euthanized via CO_2_ inhalation and decapitated. Brains were removed, rinsed in ice-cold PBS, and the olfactory bulbs and brainstem were trimmed. Following collection, tissue blocks were immediately snap-frozen in liquid nitrogen and subsequently transferred to a −80 °C freezer for long-term storage. For sectioning, blocks were embedded in OCT compound and cut into 15 µm parasagittal sections using a cryostat. Sections were mounted on nuclease-free slides, and the CSF-contacting nucleus was collected into lysis buffer using a laser capture microdissection system (Zeiss, Jena, Germany), followed by storage at −80 °C.

### 4.12. Quantitative PCR

Following cDNA synthesis from total RNA with a commercial kit (ABclonal, Woburn, MA, USA, RK20429), qPCR was carried out utilizing SYBR Green Master Mix (ABclonal, RK21203) on a QuantStudio 7 Flex instrument (Thermo Fisher Scientific, Waltham, MA, USA). GAPDH was used as the endogenous control for normalization, and relative mRNA expression was calculated via the 2−ΔΔCt method. Melt curve analysis was performed at the end of each qPCR run to verify amplification specificity and exclude nonspecific products or primer-dimer formation. Primers were designed to span exon–exon junctions where possible to minimize genomic DNA amplification. Primer sequences and corresponding reference sequence accession numbers are listed in Table 1.

### 4.13. Statistical Analysis

GraphPad Prism 8.0 was employed for all statistical computations, with experimental values depicted as mean ± SEM. Between-group differences were assessed using unpaired two-tailed *t*-tests (two groups) or one-/two-way ANOVA with appropriate post hoc tests (multiple groups), with a *p* value < 0.05 deemed statistically significant.

## 5. Conclusions

In summary, this study establishes the CSF-contacting nucleus as a central hub that links neuroinflammation to depressive-like behaviors, and identifies the PSMB4/MHC-I signaling axis as a critical molecular mediator. Collectively, our results shed light on the inherent immune mechanism of depression neurons and identify potential targets for treatment interventions for inflammatory-related mood disorders.

## Figures and Tables

**Table 1:** Primers sequence for RT-qPCR.

Gene	Accession No.	Sequence (from 5′ to 3′)	Tm (°C)
*PSMB4*	NM_008945.3	CCCATCACTCGGACCCAGAAC	63.7
		AGCCAGGGAGCCGTAGGAG	61.5
*MHC-I*	NM_153749.4	CTACGACGGCTGCGATTACATC	62.4
		GCCTGCTCCCACTTGTGTTTG	62.8
*GAPDH*	NM_008084	AGGTCGGTGTGAACGGATTTG	62.1
		GGGGTCGTTGATGGCAACA	61.9

## Data Availability

All data are included in this article, with additional datasets available from the corresponding author upon reasonable request post-publication.

## Supplementary Materials

The following supporting information can be downloaded at: https://www.mdpi.com/article/10.3390/ijms27114798/s1.

## Abbreviations

The following abbreviations are used in this manuscript:

AAV	Adeno-associated virus
Aq	Cerebral aqueduct
CB	Cholera toxin B
CBB	Cerebrospinal fluid–brain barrier
CB-HRP	Cholera toxin subunit B conjugated with horseradish peroxidase
CB-SAP	Cholera toxin subunit B conjugated with saporin
CNO	Clozapine N-oxide
CNS	Central nervous system
Cre	Cyclization recombination enzyme
CSF	Cerebrospinal fluid
CSF-contacting	Cerebrospinal fluid-contacting nucleus
Nucleus	Nucleus
Ctrl	Control
DAPI	4′6-Diamidino-2-phenylindole
FST	Forced swimming test
GAPDH	Glyceraldehyde-3-phosphate
GFAP	Glial fibrillary acidic protein
IBA1	Ionized calcium binding adaptor molecule 1
i.c.v.	Intracerebroventricular injection
IF	Immunofluorescence
IL-1β	Interleukin-1β
IL-6	Interleukin-6
LCM	Laser capture microdissection
LPS	Lipopolysaccharide
LV	Lateral ventricle
MDD	Major depressive disorder
MHC-I	Major histocompatibility complex class I
NeuN	Neuronal nuclei
NS	Normal saline
OFT	Open field test
PBS	Phosphate buffered saline
PSMB4	Proteasome 20S subunit beta 4
RT-qPCR	Real-time quantitative polymerase chain reaction
shRNA	Short hairpin RNA
SPT	Sucrose preference test
TNF-α	Tumor necrosis factor-α
TST	Tail suspension test
WB	Western blot
