# Supplementary material for: PSMB4/MHC-I Signaling in the Cerebrospinal Fluid-Contacting Nucleus Mediates Neuroinflammatory Depression in Mice
Source: Int J Mol Sci. 2026 May 26;27(11):4798. doi: 10.3390/ijms27114798 (PMC13257190; doi:10.3390/ijms27114798)
Supplement: Supplementary file 1 [file ijms-27-04798-s001.zip › ijms-4289680-supplementary.pdf]

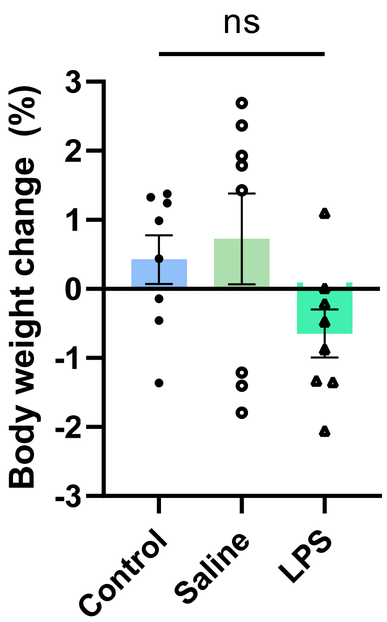

Supplementary Figure S1. Body weight changes of mice between baseline and the 72 h behavioral testing time point. The bar graph illustrates the body weight change ratio (%) of mice in the Control, Saline, and LPS groups. No significant differences in body weight changes were observed among the groups at 72 h post-injection, indicating that the acute sickness-associated systemic effects had largely subsided prior to the behavioral evaluations. n=8 per group. In the bar graph, the light blue bar represents the Control group, the light green bar represents the Saline group, and the cyan bar represents the LPS group. Data are presented as mean ± SEM. ns, not significant ($P > 0.05$, evaluated by one-way ANOVA).
